# Men’s knowledge, attitude, and barriers towards emergency contraception: A facility based cross-sectional study at King Saud University Medical City

**DOI:** 10.1371/journal.pone.0249292

**Published:** 2021-04-26

**Authors:** Syed Irfan Karim, Farhana Irfan, Hussain Saad, Mohammed Alqhtani, Abdulmalik Alsharhan, Ahmed Alzhrani, Feras Alhawas, Saud Alatawi, Mohammed Alassiri, Abdullah M. A. Ahmed

**Affiliations:** 1 Department of Family and Community Medicine, College of Medicine, King Saud University, Riyadh, Saudi Arabia; 2 Department of Family and Community Medicine, King Saud University Chair for Medical Education Research and Development, College of Medicine, King Saud University, Riyadh, Saudi Arabia; 3 College of Medicine, King Saud University, Riyadh, Saudi Arabia; FHI360, UNITED STATES

## Abstract

**Background:**

Male partners have a considerable role in influencing women’s contraceptive decision making to reduce the chance of unintended pregnancy. Most studies are focused on women’s knowledge and barriers for emergency contraception (EC) use. There is limited research on this topic from the male perspective. This study aimed to gather baseline data on men’s knowledge, attitudes and barriers about EC.

**Methods:**

Descriptive analytic cross-sectional study was conducted from Dec 2019 –May 2020 at the King Khalid University Hospital (KKUH); a teaching facility with general and subspecialty medical services in King Saud University Medical City (KSUMC), Riyadh, Saudi Arabia. Data were collected using a structured pretested questionnaire and analyzed using SPSS version 23.0. Descriptive statistics and Chi square tests were used. Multivariate logistic regression analysis was used to find significant predictors for EC awareness and use. A p value < 0.05 was considered statistically significant.

**Results:**

A total of 461 participants completed the questionnaire (response rate 86%). The majority (82%) of the participants were unaware of EC; with only 18% having some knowledge. Knowledgeable men had positive attitudes (73.5%) about EC as compared to non- knowledgeable ones (55.0%). Factors found to be associated with less knowledge of EC were cultural [0.46, 95%CI 0.22. 0.96] and religious unacceptability [OR 0.51, 95%CI 0.29, 0.89)]. Higher level of education [OR 1.83, 95%CI 0.94, 3.53] was associated with more knowledge regarding EC. The study showed that correct information about using contraceptives within 3 days of unprotected sex [OR 4.96, 95%CI 1.81, 13.60]; availability without prescription [OR 5.06, 95%CI 1.68, 15.30], EC advertisement [OR 4.84, 95%CI 0.96, 24.27] and receipt of information from family/friends [OR 18.50, 95%CI 5.19, 65.93] were factors that contributed to men using EC.

**Conclusion:**

The current knowledge of EC among men is limited. Social determinants affect these levels of knowledge, as well as the usage of EC. Factors that were associated with the use of ECPs were correct knowledge, advertisement, availability and receipt of information from family/friends. The findings highlight the need to educate men on this important topic to avoid unintended pregnancy, keeping in view cultural and social values. Future qualitative studies are needed to understand the male perspective.

## Introduction

Many couples desire small families and space out their children by preventing unintended pregnancies, but don’t always succeed. Worldwide, approximately 40% of all pregnancies are unintended [1, 2] of which 30%, are due to contraceptive failure [3]. It could be either due to method or user failure. Emergency contraception is a therapy used to prevent pregnancy after an unprotected or inadequately protected act of sexual intercourse [4]. Despite the many documented benefits of emergency contraception, its use continues to be less in Muslim countries, compared to the West [5, 6].

In contrast to most Muslim countries, the awareness and use in the Middle East and North Africa region (MENA) (especially the Arab world) of emergency contraception (EC) methods is even lower [7, 8]. The preferences for contraceptive methods in this region are also different than those in the West [9].

Effective contraception method use in the Muslim world is important because of the religious and cultural reasons forbidding the termination of pregnancies [10–12]. EC use is a prominent topic of debate and controversies exist that may be due to lack of knowledge and conflicting views about contraception and abortion [12]. Contraception is considered primarily a women’s issue [13]. Mirroring a common trend in contraceptive health, majority of research on EC is centered around females’ perspectives towards its usage. Although, men also have a vital role in preventing unintended pregnancies [14].

A study on Muslim womens’ positions in deciding on the use of contraceptives found that most women considered it natural to involve their partner in the decision [7]. This finding is in line with the literature on contraceptive decision making [15]. A step that men can take is to encourage their partner to use EC, as timely use may prevent unintended pregnancy by 89–95% [16].

Individuals sexual behavior and preferences are influenced by many factors at multiple levels such as personal attitude, beliefs, community gender norms and partners influence over method selection [17, 18]. While most couples do utilize a method of contraception, consistent use is affected by numerous barriers such as a dislike of method side effects, lack of information, cost of contraceptive methods, and culture [19]. This indicates that many couples may face periods of unprotected intercourse and the risk of unintended pregnancy.

It is important to consider men’s attitudes and behaviors, more specifically their level of awareness, when it comes to contraceptive use as it can either enable or inhibit contraception use and healthy sexual behaviors. There are several gaps in the literature on contraceptive use among men that analyzes the issues inherent in this process and very few studies have dealt with this topic [20]. Notably, studies that do report the male reproductive behavior about EC, limit findings to Western perspective only. The extent to which these findings can be generalized to men in other low and middle income countries is unclear. There is a pressing need to investigate males’ conceptualization of EC in other parts of the world, as unintended pregnancies and EC use is not restricted by gender or geographical boundaries.

Hence, based on the research gaps discussed above, this study was designed to explore knowledge, attitude and barriers about EC among men. In this study the Ecological Systems Theory (EST) [12]and Gender Systems Theory (GST) [21] was utilized.

EST hypothesizes that an individual’s development and behavior is defined through an interrelationship between the individual and different environments that can influence an individual response at different levels. This framework emphasizes that individual behaviors and perceptions are influenced by intra and interpersonal attributes and wider societal- community contexts [22, 23].

Gender to a great degree is involved in reproductive behaviors. A couple’s decision to use contraception is situated within a gendered system; an individual’s education, culture norms and family are a few of the determinants.

## Materials and methods

### Study design and setting

KKUH, KSUMC is a multi-disciplinary teaching facility with general and subspecialty medical services; situated in the capital city Riyadh, Saudi Arabia. It offers outpatient and inpatient facility, laboratory, radiology, and pharmacy services, a dedicated home healthcare program and free primary, secondary & tertiary care to its patients. Over a six- month period (Dec 2019- May 2020), a cross-sectional study was conducted at major outpatient clinics at the hospital. The clinics included the General out-patient Clinics; Family Medicine and several specialty clinics including Surgery, Medicine, Dermatology, Pediatrics, Orthopedics, Psychiatry, Gynecology, ENT, Eye, Oncology, Pain Clinics, Emergency Care Clinics, Dental Clinics and Physical Therapy Clinics.

### Target population/sampling

The target population were men attending the out-patient Clinics.

The Good Calculators [24] website was used to estimate the required sample size using the formula: n = (Za/2)2 p(1-p) / d2, based on previous study findings [14] which indicate that the knowledge for the emergency contraceptives among the male population ranges between 22% to 98%. We estimated the sample with the following assumption: prevalence of knowledge at 22% (a figure chosen because we did not find any prior published studies on knowledge in a similar setting) with a confidence level of 95% and a margin of error of 5%; the required sample size was estimated to be 385. Adding a 25% expected nonresponse or refusals due to the sensitive topic, the total sample size was 480 (≈500) men.

A systematic sampling technique was used to approach the participants. The researchers used a fixed pattern and selected every third person in the clinic waiting area to be included in the study. Men who were in the reproductive age group (16–60 years) and were able to speak either English or Arabic consented to participate and were enrolled. Men with any kind of mental illness or problem with understanding were excluded to decrease redundancy of information.

### Data collection

All the members of the study project were trained for the data collection process.

The instrument was initially pilot tested. Participants were screened for eligibility by the research team members. The instrument in a printed format along with a covering letter; highlighting the aims and objectives of the study was used. These were filled through personal face to face interviews with emphasis on the understanding and importance of the topic.

### Emergency contraception knowledge

For our study we defined knowledge as familiarity, awareness, or understanding of something or what a person knows. We used a faceted approach of classification as it does not require the full extent of knowledge, just the declarative (one’s point of view); thus, it is particularly useful for new and emerging topics [25, 26].

### Data collection tool

A team of three clinicians who had experience in research and six medical students reviewed the literature and a questionnaire was developed which was relevant and easy to complete. The literature search on validated inventories for the measurement of emergency contraception knowledge among men is sparse. We adapted the questions from the previously published studies [7, 27, 28]; as it was the best available option on the topic. Additionally, few questions were added to cover the male perspective and rephrased to make it easy to respond. The first draft was developed by the principal investigator and an initial review was done by the other two clinicians and students. For data quality, the English questionnaire was translated into the local language, Arabic, then re-translated to English by another translator to check the consistency of the original meaning. After the initial pilot testing, necessary modifications were made to improve the clarity and understandability of the questionnaire. The questionnaire consisted of 26 items/ statements. The first part of the questionnaire included questions on socio-demographic characteristics (age, marital status, level of education, occupational status, income, nationality) and reproductive characteristics (number of children). The second part included questions on knowledge, attitudes towards contraceptive use, and barriers/reasons for not using contraceptives.

We used a quasi-filter and framed the topic in terms of an opinion question which required a “yes”, “no”, or “don’t know” answer [29]. The topic was posed in the form of a question; “if a woman has unprotected sex, is there anything she can do in the first three days after intercourse that will prevent pregnancy”? The other question was posed in statement form; “Ever heard of EC?”. By adopting this opinion statement frame-work we hoped to reduce the threat to the respondent when the issue was unfamiliar.

The knowledge on Emergency Contraceptives was based on the participants’ response to the question: “if a woman has unprotected sex, is there anything she can do in the first three days after intercourse that will prevent pregnancy”? and question “Ever heard about EC?”. Those who answered “yes” were considered to have knowledge, while those who answered “no” were considered as not having any knowledge of EC. Those who said that they “didn’t know” or gave an ambiguous answer were also considered not to have knowledge of EC. Those who had knowledge were asked when women can use EC, what they can do to prevent pregnancy and what is the correct timing for its use? Further, their knowledge was assessed for its availability and any pre-requisites before its use and the source of their knowledge. The rest of the questions were about participants’ attitudes and perceived barriers towards EC use respectively. The questions related to barriers were classified as religion, culture, difficulty to access, drug side effects, and cost.

To assess the attitudes among participants who had no knowledge about EC, the interviewer gave them an explanation on the topic and reassured them that their answers will be kept private. Further questions were only asked once the participants agreed to answer the questions. In an attempt to make the respondent willing to voice their opinion, the interviewer read a series of statements about EC use. After each one, the participants voiced a true opinion for each.

In the administration of the scale, the attitudes were evaluated by using a “yes”, “no”, or “don’t know” format. There were ten statements given, along with the options of yes (1), no (2), don’t know (0). The lowest score obtainable was 0, whereas the highest score 10. The cutoff value was taken as “5”. A higher score (>5) was taken as positive/favorable attitude and a lower score (score = or <5) as not positive/unfavorable attitude towards EC use. The alpha coefficient for our variables was 0.50. From the social-ecological perspective [18], questions were asked to cover the following four domains:

*Intrapersonal level*: characteristics of the individual such as demographics, knowledge, attitudes, behavior, perceptions.
*Interpersonal level*: with whom the at-risk people associate like family, friends; partner’s approval and involvement in contraception use.
*Organizational level*: health care infrastructure, access to health care.
*Community level*: common values and mutual concern, culture, gender norms, barriers, information available about family planning.

### Data analysis

The Statistical Package for the Social Sciences (SPSS) [version 23] was used for data analysis. Descriptive statistics such as frequency and percentage were calculated for socio-demographic data, sources of ECP education and responses to questions about ECP knowledge. The Chi-square test was used to test the association and estimate the statistical significance.

The univariate analyses were computed using descriptive statistics. To identify factors that may be associated with the awareness and utilization of EC, we employed multivariate logistic regression. For the analysis of factors affecting its usage, we used the number of participants with the correct information about using contraceptive within 3 days of un-protective sex. P-values less than 0.05 were considered statistically significant.

### Ethics approval and consent to participate

Ethical permission for the study was obtained from the Institutional Review Board of the College of Medicine; KSU (Research Project no E- 19–4404 dated 08-12-2019). Informed verbal consent was obtained from all participants by informing them that participation was voluntary and by completing the survey, they were giving consent to participate in this study. The Institutional Review Board determined that this was adequate for obtaining informed consent and waived the requirement for written consent. Anonymity and confidentiality were assured. Participants were given assurance that the information gathered will be used exclusively for research purposes.

## Results

### Socio-demographics characteristics

A total of 461 participants completed the questionnaire (response rate 86%). Table 1 summarizes the socio-demographic characteristics of the respondents. Participant age ranged from 18 to 70 years, with an overall mean of 38.2 years (SD 11.3). The majority (78.5%) were currently married, of Saudi background (96%, n = 443); having less than two children (53%, n = 243), employed (84%, n = 389) and earning > 2700 USD monthly (53%, n = 244). The median level of education was university and above.

**Table 1:** Socio-demographic characteristics of the study population (N = 461).

Variables		n (%)
Age	Mean age in Years **38.25 ± 11.3**	
	<38	255 (55.6%)
	>38	206 (44.6%)
Marital Status	Married	362 (78.5%)
	Unmarried	99 (21.4%)
Number of Children	Less than Two	243 (52.7%)
	More than Two	218 (47.3%)
Current Desire for Children	Yes	267 (57.9%)
	No	194 942.1%)
Nationality	Saudi	443 (96.1%)
	Non- Saudi	18 (3.9%)
Level of Education	High School	115 (25%)
	University	346 (75%)
Occupational Status	Employed	389 (84.4%)
	Unemployed	72 (15.6%)
Monthly Income	less than SR 10,000	217 (47.1%)
	more than SR 10,000	244 (52.9%)

### Key social determinants affecting knowledge and use of EC

#### Intra personal-level influences on contraceptive use

Awareness of the existence of EC was very low (18%, n = 83), with 82% (n = 378) of men never having heard of EC prior to the study. Men who were young, those with a higher education level, those with a lesser desire for children, and those with a few number of children were more likely to have knowledge of EC. A statistically significant association was found between knowledge and age (P = 0.04), level of education (p = 0.03), number of children (p = 0.02) and desire for children (p = 0.04) (Table 2).

**Table 2:** Difference in characteristics of men with or without knowledge about emergency contraception.

Variables	Category	Knowledge	No Knowledge	p- Value
		(83)	(378)	
		(n, %)	(n, %)	
Age	< 38	54 (65.1)	201 (53.2)	0.049
	>38	29 (34.9)	177 (46.8)	
Number of Children	Less than 2	53 (63.9)	190 (50.3)	0.025
	More than 2	30 (36.1)	188 (49.7)	
Level of Education	Schools	13 (15.7)	102 (27)	0.031
	University	70 (84.3)	276 (73)	
Monthly Income	Less than 10,000SR	35 (42.2)	182 (48.1)	0.323
	More than10,000SR	48 (57.8)	196 (51.9)	
Occupational Status	Employed	70 (83.3)	319 (84.4)	0.990
	Un employed	13 (15.7)	59 (15.6)	
Desire for Child	Yes	40 (48.2)	227 (60.1)	0.047
	No	43(51.2)	151 (39.9)	
Marital Status	Married	60 (72.3)	302 (79,9)	0.127
	Unmarried	23 (27.7)	76 (20.1)	
Nationality	Saudis	79 (95.2)	364 (96.3)	0.635
	No Saudis	4 (4.8)	14 (3.7)	

### Knowledge of men about contraceptive methods

**Fig 1:**
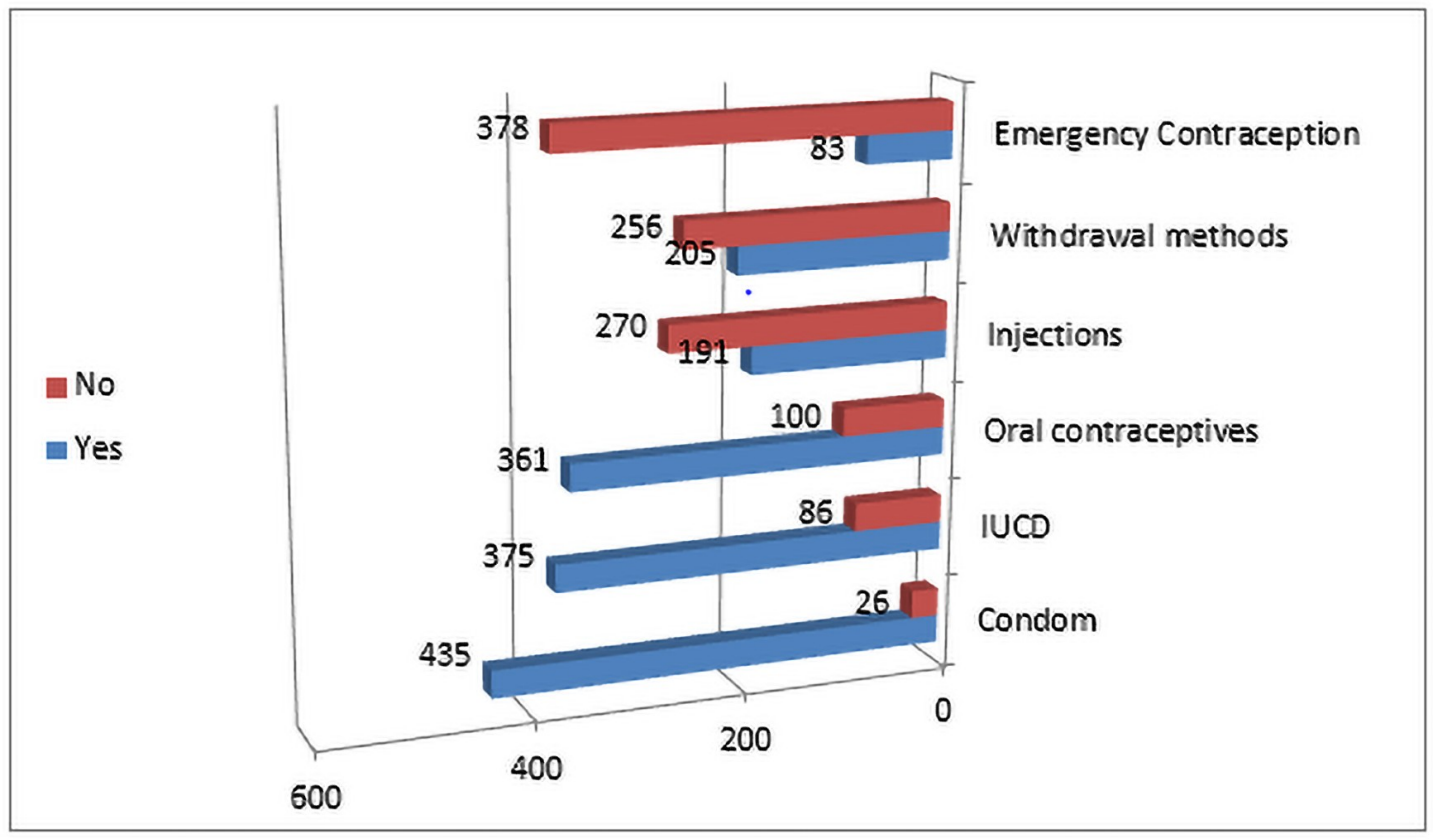
Men’s knowledge about different methods of contraception.

The most common contraceptive method named by the participants was the male condom (435/461, 94%), with the next most common being implants intrauterine device (375/461, 81%) and birth control pills (361/461, 78%). A little less than half the participants mentioned periodic abstinence and injectable hormonal contraception (205/ 461, 45%; 191/461, 41%), and very few (83/461,18%) had heard about EC (Fig 1).

Among the men who had awareness (18%), about 52% of participants correctly identified EC as effective in preventing pregnancy following unprotected intercourse.

The correct timeframe of effectiveness of emergency contraceptive pill (ECP) (up to 72 hours after unprotected intercourse) was identified by sixty percent (n = 50). Out of 83 men who were aware of ECP, only 25 (30%) had used ECP. Majority thought that a pregnancy test is necessary (71%, N = 59) and physician consultation is needed (86%, n = 71) before using ECP. Missed contraceptive pills (49%), failure of barrier contraceptives/ withdrawal method (36%) and condoms breaking during intercourse (15%) were chosen as appropriate situations for ECP use (Table 3).

**Table 3:** Knowledge of participants about EC among men (N = 83).

Response	Respondents	N = 83 (%)	
Is pregnancy test required before ECP pill?	Yes	59 (71.1)	
	No	24 (28.9)	
Do you need to consult a doctor before using ECP?	Yes	71 (85.5)	
	No	12 (14.5)	
Is ECP available in the Market?	Yes	55 (66.3)	
	No	28 (33.7)	
Have you ever used ECP to prevent pregnancy in the past?	Yes	25 (30.1)	
	No	58 (69.9)	
What is the correct timing of ECP?	< 72 hours	50 (60.2)	
	>72 hours	4 (4.8)	
	Don’t know	29 (34.9)	
What is the source of knowledge about ECP?	Magazine	Yes	No
		7 (8.4)	76 (91.6)
	Friend	13 (15.7)	70 (84.3)
	Family member	17 (20.5)	66 (79.5)
	TV	13 (15.7)	70 (84.3)
	Doctor or FP provider	26 (31.3)	57 (68.7)
	Internet	20(24.1)	63(75.9)
What can you ask her to do to prevent pregnancy?	Ask the wife to take extra birth control pill	12 (14.5)	
	Ask the wife to use emergency contraception	47 (56.6)	
	Ask the wife to have an Intra Uterine Device (IUCD) inserted	22 (26.5)	
	Ask the wife to have an abortion	(0%)	
	Ask the wife to use herbal remedies	(0%)	
	Pray	2 (2.4)	
Why would you use ECP?	To prevent Abortion	2 (2.4)	
	To prevent unwanted pregnancy	43 (51.8)	
	For Birth Spacing.	38 (45.8)	
When women can use ECP?	Condom Break.	12 (14.5)	
	Forget to take Pill.	41 (49.4)	
	Failed withdrawal ejaculation.	26 (31.3)	
	Failure to use barrier methods.	4 (4.8)	

### Men attitudes, beliefs and barriers towards emergency contraception

Regarding the level of attitude, 269 men (58.4%) had favorable/positive attitudes towards ECPs. Participants’ attitudes and barriers towards ECP have been analyzed. The attitude for all individual questions were significantly higher (positive) for knowledgeable than non- knowledgeable men (Table 4).

**Table 4:** Participants attitudes and beliefs regarding emergency contraception (n = 461).

Domain	Statement	Knowledge N = 83 (%)	No knowledge N = 378 (%)	Total N = 461 (%)	p value
Affective (Feelings)	The decision to use ECP is ultimately the decision of;				0.047
	Male/female partner	5 (6%)	53 (14%)	58(12.5)	
	Both	78 (94%)	325 (86%)	403(87.4)	
	Should ECP be more widely advertised?				0.132
	Yes	57 (68.7)	226 (59.8)	283(61.3)	
	No	26(31.3)	152 (40.2)	178(38.6)	
	Should ECP be available without prescription?				0.023
	Yes	28 (33.2)	83 (22)	111(24.0)	
	No	55 (66.3)	295 (78)	350(76.0)	
	Would you prefer your partner to get them ECP from the pharmacy or clinic?				0.034
	Yes	61 (73.5)	231 (61.1)	292(63.3)	
	No	22 (26.5)	144 (38.9)	166(36.0)	
	Men should be able to buy ECP				0.016
	Yes	69 (83.1)	265 (71.1)	334(72.5)	
	No	14 (16.9)	113 (29.9)	127(27.5)	
Behavioral (Behavior)	ECP reduces the chance of pregnancy by up to 75%, would you ask your wife to use it to prevent pregnancy?				0.001
	Yes	56 (67.5)	177 (46.8)	233(50.5)	
	No	27 (32.5)	201 (53.2)	228(49.4)	
	I would buy EC to have at home or on hand, just in case of emergencies				0.007
	Yes	60(72)	213(56)	273(59.2)	
	No	23(28)	165(44)	188(40.8)	
	Men being able to buy ECP would help to prevent unplanned pregnancies.				0.000
	Yes	71(85. 5)	229 (60.6)	300(65.1)	
	No	12 (14.5)	149 (39.4)	161(34.9)	
	I’d recommend ECP to a man at risk of being involved in an unplanned pregnancy.				0.000
	Yes	71 (85.5)	229 (60.6)	300(65.0)	
	No	12 (14.5)	149 (39.4)	161(35.0)	
Cognition(Beliefs)	Do you feel embarrassed to buy?				0.076
	Yes	16 (19.3)	109 (28.8)	125 (27.1)	
	No	67(80.7)	269 (71.2)	336 (72.9)	
	What are the reasons for not using EC?				
	Religion				0.010
	Yes	18 (21.7)	138 (36.5)	156(33.8)	
	No	65 (78.3)	240 (63.5)	305(66.1)	
	Culture				0.039
	Yes	9 (10.8)	78 (20.6)	87(18.8)	
	No	74 (89.2)	300 (79.4)	374(81.1)	
	Difficulty to access				0.532
	Yes	19 (22.9)	75 (19.8)	94(20.3)	
	No	64 (77.1)	303 (80.2)	367(79.6)	
	Drug Side Effects (Nausea, Vomiting etc.)				0.386
	Yes	37 (44.6)	149 (39.4)	186(40.3)	
	No	46 (55.4)	229 (60.6)	275(59.6)	
	Cost				0.051
	Yes	14 (16.9)	36 (9.5)	50(10.8)	
	No	69 (83.1)	342 (90.5)	411(89.1)	
Overall Attitude scores	Positive/favorable attitude (score >5)	61(73.5)	208(55.0)	269(58.4)	0.002
	Not so positive/unfavorable attitude (score = or <5)	22(26.5)	170(45.0)	192(41.6)	

Among the total participants, 87% (n = 403) reported that they would mutually discuss it with their partners before beginning usage. Overall, majority (73%, n = 336) were not shy to ask for ECP in case of need. More than half (69%) of the knowledgeable men were of the opinion that ECP should be freely available over the counter, without prescription in order to avoid unwanted pregnancy. This difference was statistically significant (p = 0.02). When asked about their preference to use ECP if it reduces the chance of pregnancy by 75%, majority of them were in favor (p = 0.001). Men who had knowledge were also in favor of buying it and having it on hand prior to an episode of contraceptive failure (p = 0.01), preferred their partners to get the pill from the pharmacy/clinic (p = 0.03), and were in favor of advertising ECP (p = 0.08). They would also recommend it to other men at risk (p = 0.000) (Table 3).

### Interpersonal-level factors

Assessing the different sources of knowledge about ECP yielded mixed results (Tables 3 and 4). It was also found that group conversations (friends / family members) were the main sources of knowledge (36.2%, n = 30) followed by physicians /family planning providers (31.3%, n = 26), internet /mass media (24%, n = 20) and magazines (8.4%, n = 7).

#### Organizational and community level factors

Majority (66%, n = 55) of men knew that ECP was available at pharmacies and were in favor of their partners (63.3%, n = 292) and themselves (72.4%, n = 334) having access to pharmacies to get it if the need arose.

### Barriers to ECPs use

The main reasons deterring men not to use EC were medicine side effects (40%), fear of religion (34%), difficulty to access (20.3%), culture (19%) and cost (11%) (Table 4). A significant association was found between the religion, culture, cost and the presence of knowledge (*p* = 0.01, p = 0.03, 0.05), respectively.

#### Factors/Predictors associated with the knowledge and use of EC among men

The univariate and multivariate logistic regression analysis was carried out to find the significant predictors for knowledge of ECP (Table 5). The results indicated that men who reported religion as a barrier were 49 times [OR 0.51, 95%CI 0.29, 0.89] less likely to have the knowledge about ECP. Similarly, those reporting culture as a barrier were 54 times [OR 0.46, 95%CI 0.22, 0.96)] less likely to have knowledge about ECP. Educational status was marginally significant, with university level men being [OR 1.83, 95%CI 0.94, 3.54] more likely to have the knowledge about ECP compared to school level. The association was significant after adjusting for age, marital status and occupation.

**Table 5:** Factors affecting the knowledge of ECP among men.

Variable	Knowledge (83)	No Knowledge (378)	Unadjusted odds ratio 95% confidence interval	Adjusted odds ratio 95% confidence interval	P value only for multivariate
	N (%)	N (%)			
Age (in years)					0.33
<38	54 (65.1)	201 (53.2)	1	1	
>38	29 (34.9)	177 (46.8)	0.61 (0.37, 1.00)	0.76 (0.43, 1.33)	
Educational Level					0.07
School	13 (15.7)	102 (27)	1		
University	70 (84.3)	276 (73)	1.99 (1.06, 3.75)	1.83 (0.94, 3.53)	
Marital status					0.32
Married	60 (72.3)	302 (79,9)	1		
Unmarried	23 (27.7)	76 (20.1)	1.64 (0.94, 2.86)	0.72 (0.38, 1.38)	
Occupational status					0.66
Unemployed	70 (83.3)	319 (84.4)	1		
Employed	13 (15.7)	59 (15.6)	0.99 (0.52, 1.91)	0.85 (0.41, 1.76)	
Religion as a barrier					0.02
No	65 (78.3)	240 (63.5)	1		
Yes	18 (21.7)	138 (36.5)	0.48 (0.27, 0.84)	0.51 (0.29, 0.89)	
Culture as a barrier					0.04
No	74 (89.2)	300 (79.4)	1		
Yes	9 (10.8)	78 (20.6)	0.49 (0.22, 0.97)	0.46 (0.22, 0.96)	

Logistic regression analysis was carried out to find the significant predictors for utilization of EC (Table 6). Presented in Table 7 are the results of multivariate analysis of factors affecting the use of ECPs after adjusting for the effects of other variables (age, education and barriers to ECP). Correct information about the time frame for ECP use [OR 4.96, 95%CI 1.81, 13.60], availability of ECPs (OR 5.06, 95%CI 1.68, 15.30], its wide advertisement [OR 4.84, 95%CI 0.96, 24.27] and friends and family as source of information [OR 18.50, 95%CI 5.19, 65.93] remained significant predictors of the use of ECPs.

**Table 6:** Univariate analysis for the factors of ECP use among men.

Variable	Have used ECP = 25 (5.4)	Have not used ECP = 436 (94.6)	Unadjusted odds ratio (95% CI)	P value
Correct information about using contraceptive within 3 days of un-protective sex				<0.001
No	11 (44.0)	372 (85.3)	1	
Yes	14 (56.0)	64 (14.7)	7.39 (3.22, 17.02)	
Age				0.628
< 38	15 (60)	240 (55)	1	
>38	10 (40)	196 (45)	0.82 (0.36, 1.86)	
Level of Education				0.717
Schools	7 (28.0)	108 (24.8)	1	
University	18 (72.0)	328 (75.2)	0.85 (0.34, 2.08)	
Number of children				0.735
<2	14 (56.0)	229 (52.5)	1	
>2	11 (44.0)	207 (47.5)	0.87 (0.38, 1.95)	
Should ECP be more widely advertised?				0.001
No	2 (8.0)	176 (40.4)	1	
Yes	23 (92.0)	260 (59.6)	7.78 (1.81, 33.43)	
Should ECP be available without prescription?				<0.001
No	9 (36.0)	341 (78.2)	1	
Yes	16 (64.0)	95 (21.8)	6.38 (2.73, 14.89)	
Would you prefer your partner to get the ECP from the pharmacy or clinic?				0.619
No	8 (32.0)	161 (36.9)	1	
Yes	17 (68.0)	275 (63.1)	1.24 (0.525, 2.947)	
Men should be able to buy ECP				0.184
No	4 (16.0)	123 (28.2)	1	
Yes	21 (84.0)	313 (71.8)	2.06 (0.69, 6.13)	
ECP reduces the chance of pregnancy by up to 75%, would you ask your wife to use it to prevent pregnancy?				0.003
No	5 (20)	223 (51.1)	1	
Yes	20 (80)	213 (48.9)	4.18 (1.54, 11.35)	
Men being able to buy ECP would help to prevent unplanned pregnancies.				0.016
No	3 (12.0)	158 (36.2)	1	
Yes	22 (88.0)	278 (63.8)	4.17 (1.23, 14.14)	
I’ll recommend ECP to a man at risk of being involved in an unplanned pregnancy.				0.002
No	2 (8.0)	161 (36.9)	1	
Yes	23 (92)	275 (63.1)	6.73 (1.56, 28.93)	
Do you feel embarrassed to buy?				0.665
No	16 (64.0)	260 (59.6)	1	
Yes	9 (36.0)	176 (40.4)	0.83 (0.35, 1.92)	
What are the reasons for not using EC?				0.133
Religion				
No	20 (80)	285 (65.4)	1	
Yes	5 (20)	151 (34.6)	0.47 (0.17, 1.28)	
Culture				1
No	21 (84.0)	353 (81.0)	1	
Yes	4 (16.0)	83 (19.0)	0.81 (0.27, 2.42)	
Difficulty to access				0.332
No	18 (72.0)	349 (80.0)	1	
Yes	7 (28.0)	87 (20.0)	1.56 (0.63, 3.85)	
Side effects				0.702
No	14 (56.0)	261 (59.9)	1	
Yes	11 (44.0)	175 (40.1)	1.17 (0.52, 2.64)	
Cost				0.849
No	22 (88.0)	389 (89.2)	1	
Yes	3 (12.0)	47 (10.8)	1.129 (0.32, 3.914)	
Source of Information				<0.001
Doctor				
No	15 (60)	420 (96.3)	1	
Yes	10 (40)	16 (3.7)	17.50 (6.81, 44.94)	
Internet/Magazine/TV				<0.001
No	13 (52.0)	412 (94.5)	1	
Yes	12 (48.0)	24 (5.5)	15.48 (6.53, 38.43)	
Friends/family				<0.001
No	16 (64.0)	417 (95.6)	1	
Yes	9 (36.0)	19 (4.4)	12.34 (4.84, 31.51)	

**Table 7:** Multivariate analysis for the factors for ECP use among men.

Variable	Have used ECP = 25 (5.4)	Have not used ECP = 436 (94.6)	Unadjusted odds ratio (95% CI)	Adjusted odds ratio	95% confidence interval	P value
Correct information about using contraceptive within 3 days of un-protective sex						0.002
No	11 (44.0)	372 (85.3)	1	1		
Yes	14 (56.0)	64 (14.7)	7.39 (3.22, 17.02)	4.96	1.81, 13.60	
Should ECP be more widely advertised?						0.05
No	2 (8.0)	176 (40.4)	1	1		
Yes	23 (92.0)	260 (59.6)	7.78 (1.81, 33.43)	4.84	0.96, 24.27	
Should ECP be available without prescription?						0.004
No	9 (36.0)	341 (78.2)	1	1		
Yes	16 (64.0)	95 (21.8)	6.38 (2.73, 14.89)	5.06	1.68, 15.30	
Friends/family						<0.001
No	16 (64.0)	417 (95.6)	1	1		
Yes	9 (36.0)	19 (4.4)	12.34 (4.84, 31.51)	18.5	5.19, 65.93	

Adjusted for age, education, and barriers to use ECP

## Discussion

This is the first study on knowledge, attitude, barriers and use of ECPs as well as factors associated with it among men in the Arab region. The study found an alarmingly low level of knowledge and understanding among Saudi men. As theoretically expected, our findings indicated that participants’ affective and cognitive beliefs were significantly associated with intentions to use, highlighting its importance over the behavior.

Studies investigating gender differences have confirmed that knowledge of EC was lower among men than their counterparts [14]. However, it is not surprising that males know less than females about emergency contraception as reproductive health policies such as marketing strategies, counselling, and in general dissemination of knowledge regarding fertilization and contraception continue to be more focused on women [14].

With respect to the knowledge of EC, it is clear that the men knew little about its indications and effectiveness. Although they did have positive attitudes about emergency contraception, improvements in their knowledge for the use of the method is needed.

This study conjures a snapshot of the factors that can influence the contraceptive knowledge and use. Studies have shown that gaps in contraceptive knowledge could be explained by the key social determinants [7, 30]. A significant association between knowledge and level of education, culture, and religious beliefs was found and is consistent with studies done in the Arab region with similar cultural background [31–34]. Higher level of education was associated with more knowledge of ECP and more positive attitudes towards actual use.

The study findings indicate that religious and cultural values were the source of knowledge proscriptions for many individuals. A probable explanation could be that individuals who value religion and culture in their lives (have greater religious commitment) are more likely than others to not indulge themselves in seeking knowledge of contraception and accept religious doctrines.

Further, our findings indicate that older men were less knowledgeable about EC than younger men, probably because the younger generation is better educated, more technologically savvy, has greater exposure to social media and is more open to talking about sex. This is in line with a recent assessment which found that knowledge of EC among men varied according to age, location and community setting [14].

An overall pattern of partial knowledge was observed throughout the responses. Inadequate knowledge about contraceptive methods has been cited as one of the major reasons for the lack of its use [7], and knowledge had a statistical effect on EC use in our population. The participants were familiar with at least one modern contraceptive method, with the condom being the most commonly known method and EC the least. The higher familiarity of condoms is in line with reported studies which found that men focus primarily on condom use and see this as a male partner’s only available contraceptive contribution [16]. This illustrates a probable lack of male participation in reproductive health discussions. In contraceptive socialization, the most important sources of information on EC in this study were friends/family members followed by physicians /family planning providers. Further confirming our findings, logistic regression analysis indicated that participants whose source of information were family/friends were more likely to use it. We assume that men felt it easy to discuss this topic with people from their social network, most likely another man close enough to them that they felt confident in asking about contraceptive matters. This finding is consistent with local studies [7, 35, 36]. Our findings highlight the need to include counseling as group sessions with men of common beliefs as it may provide an opportunity for accurate information to be disseminated to the group at large. It is both concerning and unfortunate, as health care providers can be a source of reliable information related to EC. As reported in literature [37], male physicians tend to not offer discussion on contraceptive methods. Research is needed to ascertain whether or not providers themselves have enough knowledge and are happy to counsel men about EC. Previous studies have shown that physicians were unsatisfied with their current knowledge of EC and felt uncomfortable due to inexperience with its use. They also felt it an inappropriate topic to be discussed at routine consultations due to religious/ethical/cultural reasons as it could promote promiscuity [22, 38]. Unwillingness at the provider level can have significant implications as couples might not be able to obtain timely information to initiate contraception. Increasing conversations between providers and male patients may positively affect both mens’ knowledge and their comfort. Our findings call for future research on understanding the socio-cultural underpinnings of health behaviors, motivations, clinicians’ counseling and behaviors of involving males in provision of EC.

However, religion is embedded in all global cultures and can have a sizable impact on couples’ adoption of EC. There have been significant research efforts on womens’ perspectives globally but the male perspective on contraception is an underrepresented issue in the scientific research field. Only a few studies have been conducted with dissimilar cultural settings [39]. The results of this study revealed that knowledge of EC among Saudi men was much lower than the studies in other countries [19, 40, 41]. However, it was similar to a study published among Turkish men [40, 42]. This similarity could be due to religion, although, cultural backgrounds differ significantly from one society to the other [43, 44].

Much to our surprise, religious and cultural convictions were not strong imperatives for men to not use contraception. This can be a result of modernization and transformation in Saudi Arabia, akin to what has been seen in other nations. Another possibility could be due to the notion that using it is allowable in comparison to abortion, which is not allowed in Islamic prophetic text (Quran and Sunnah).

Arab culture promotes a patriarchal society where men determine most of the family issues. A study in an Arab country has shown that contraceptive use is increased many folds with the husband’s approval [33]. Contraceptive responsibility has shifted from individuals to couples and is no longer gendered labor. This study revealed that men supported mutual decisions for contraception. They expressed a desire for wide advertisement and easy availability/ purchase of ECPs which is contrary to their counterparts, a finding reported by others [7, 45]. This dissociation needs further exploration. A future qualitative study can identify the hidden concerns.

There was broad acceptance of using EC once the method and its effectiveness was explained. This shows system-related barriers such as the lack of appropriate information and minimal educational resources available in the native Arabic language [46]. In line with other studies, men were willing to recommend it to others at risk and use it in the future if the need arises [20]. This indicates that being well informed is a prerequisite for effective action and desired outcomes [47].

In light of this finding, educational leaflets and awareness sessions can be provided as part of reproductive health counseling, as reported in the literature [30, 48]. Moreover, contraception knowledge remains a sensitive religious and cultural issue with a raft of challenges. Thus, religious leaders should be involved as they can also act as agents of change. Involving them can bring additional attitude change in the social norms and practice.

## Strengths and limitations

To our knowledge, this is the first study of its kind in the Kingdom of Saudi Arabia and it provides important evidence that may be used as a baseline for further educational campaigns. Due to rapid economic and informational revolution, we anticipate a more positive change in future.

This was a cross sectional study conducted in only one of the prominent hospitals in the capital city of Saudi Arabia, covering a small group. Hence, it is not representative for all men and cannot establish causality. Therefore, generalizing the results of this study to other settings must be done with caution. Due to the sensitive nature of the topic, the respondents’ honesty and disclosure may be constrained. However, we tried to minimize this through building a good rapport by ensuring confidentiality and privacy.

Another limitation of our study is the low reliability of the questionnaire; which could be due to a relatively small sample size and the fact that we used items from existing questionnaires, as there were no reliable gold-standard contraceptive questionnaires to measure the individual’s knowledge, attitude and barriers. The current questionnaire includes only two items to measure knowledge and an opinion format was used to elicit information. Future assessments could include more items to assess knowledge. Additionally, the questionnaire in the current study addressed the barriers generally without classification into psychosocial, physical, medical, administrative and cultural aspects. Future clarification qualitative studies are needed to explore the barriers and concerns regarding the use of ECP in depth.

A larger sample size and a broader geographical distribution is recommended for further studies to gain more insight on this important reproductive health subject.

## Conclusion

The level of knowledge and awareness about EC among Saudi men was very low. Poor knowledge and EC use is affected by inter/intra-personal and socio-cultural factors. The major barriers identified were concerns about the possible side effects of EC. The findings highlight the need to educate men on this important topic in order to avoid unintended pregnancy, keeping in view culture and social values. Efforts to improve affective, cognitive, and partner-specific dimensions are promising directions for future improvements. Health care professionals can play a vital role. Future qualitative studies are needed to understand men’s perspective.

## Recommendations and implication statement

The findings of the study reveal some important policy implications that are useful for health authorities to take up the challenge of improving the quality of reproductive health services within Saudi Arabia.

Our findings regarding the importance of a social network also highlights that it may be beneficial to consider group counseling sessions with men of similar demographic backgrounds, which can provide an opportunity for accurate information to be disseminated to the group. This would increase the likeliness of relaying this information to other men. Strategies need to be undertaken to develop community-based, confidential and friendly clinic services which can offer reproductive care and planned parenthood. Health care professionals can provide appropriate counseling tailored to social norms and religious values. Larger surveys can be used to assess knowledge and dispel any myths and misconceptions.

## Supporting information

S1 Fig (XLS)

S1 Data (XLSX)

S1 File (DOCX)

S2 File (DOC)

## Abbreviations

EC: Emergency contraception
