# Correction: Men’s knowledge, attitude, and barriers towards emergency contraception: A facility based cross-sectional study at King Saud University Medical City

**DOI:** 10.1371/journal.pone.0303464

**Published:** 2024-05-02

**Authors:** Syed Irfan Karim, Farhana Irfan, Hussain Saad, Mohammed Alqhtani, Abdulmalik Alsharhan, Ahmed Alzahrani, Feras Alhawas, Saud Alatawi, Mohammed Alassiri, Abdullah M. A. Ahmed

The sixth author’s name is spelled incorrectly. The correct name is: Ahmed Alzahrani. The correct citation is: Karim SI, Irfan F, Saad H, Alqhtani M, Alsharhan A, Alzahrani A, et al. (2021) Men’s knowledge, attitude, and barriers towards emergency contraception: A facility based cross-sectional study at King Saud University Medical City. PLoS ONE 16(4): e0249292. https://doi.org/10.1371/journal.pone.0249292
