# Supplementary material for: Men’s knowledge, attitude, and barriers towards emergency contraception: A facility based cross-sectional study at King Saud University Medical City
Source: PLoS One. 2021 Apr 26;16(4):e0249292. doi: 10.1371/journal.pone.0249292 (PMC8075244; doi:10.1371/journal.pone.0249292)
Supplement: S1 File — (DOCX) [file pone.0249292.s003.docx]

**"ماذا يعرف الرجال عن وسائل منع الحمل الطارئ؟ دراسة استقصائية عن المعرفة والمواقف والحواجز التي تحول دون استخدامها"**

**اسم الباحث الرئيسي**: الدكتور سيد عرفان كريم ، أستاذ مساعد.

**عنوان المكتب :**

قسم طب الأسرة والمجتمع

كلية الطب

ص.ب 2925 ، الرياض 11461

تليفون: 4670836 – 4677953

الجوال: 0565046182

**نموذج الموافقة**

**عزيزي المشارك:**

هذا الاستطلاع جزء من دراسة تتناول "**ماذا يعرف الرجال عن وسائل منع الحمل في حالات الطوارئ؟ دراسة استقصائية عن المعرفة والمواقف والحواجز التي تحول دون استخدامها** ". الاستبيان هو تقرير ذاتي ومجهول.

يعتبر منع الحمل الطارئ خيارًا مهمًا للأزواج الذين يرغبون في تجنب الحمل في حالة فشل طريقة تنظيم الأسرة أو ممارسة الجنس دون وقاية. ومع ذلك ، بسبب نقص المعرفة والمعلومات حول استخدام حبوب منع الحمل في حالات الطوارئ ، فهي غير مستغلة بشكل كاف. من المهم استكشاف معرفة ومواقف الرجال فيما يتعلق بمنع الحمل في حالات الطوارئ ، وكذلك تحديد العوائق التي تحول دون استخدامه. ستساهم المعلومات بشكل كبير في التدخلات اللازمة لنشر المعلومات لمكافحة الحمل غير المقصود.

يشير تبادل المعرفة الطبية إلى تبادل المعلومات والمشورة والأفكار والتقارير والاكتشافات العلمية مع الأطباء الآخرين في المجتمع الطبي.

سيكون ردك موضع تقدير كبير في هذا الموضوع الهام.

مشاركتك في هذه الدراسة البحثية تطوعية.

ردودك ستكون سرية.

سيتم استخدام نتائج هذه الدراسة للأغراض العلمية فقط.

**الوقت المقدر لاستكمال الاستبيان: 7 - 10 دقائق**

**ماذا يعرف الرجال عن وسائل منع الحمل الطارئ؟**

**دراسة استقصائية عن المعرفة والمواقف والحواجز التي تحول دون استخدامها**

**استطلاع**

- العمر (سنوات):
- الحالة الاجتماعية:

□ متزوج □ غير متزوج □ مطلق

- عدد الأطفال:

□ لا يوجد □ واحد □ اثنين □ أكثر من 2 □ أكثر من 5

- الرغبة الحالية للانجاب:

□ نعم □ لا

- الجنسية:

سعودي □ غير سعودي □

- مستوى التعليم :

 ابتدائي □ متوسط □ ثانوي □ جامعى □ ما فوق الجامعى □

- حالة الوظيفة:

  حكومية □ عمل حر □ متقاعد □ □ لا أعمل

- الراتب:

أقل من 5000 ريال □ 5000 إلى 10000 ريال □ أكثر من 10 آلاف ريال □

**المعلومة:**

1. **هل سبق لك أن سمعت عن أي وسائل منع الحمل التالية؟**

|  | الواقي الذكري | جهاز اللولب الرحمي | وسائل منع الحمل عن طريق الفم | وسائل منع الحمل عن طريق الحقن | طريقة السحب | وسائل منع الحمل في حالات الطوارئ |
| --- | --- | --- | --- | --- | --- | --- |
| نعم |  |  |  |  |  |  |
| لا |  |  |  |  |  |  |

2. **إذا كان الرجل قد مارس الجماع ، فهل هناك ما يمكنه فعله في أول 3 أيام بعد الجماع لمنع الحمل؟**

   □ نعم □ لا □ لا أعرف

3. **هل سمعت عن وسائل منع الحمل الطارئ؟ (إذا كانت الاجابة نعم للسؤال 1)**

   □ نعم □ لا

**إذا كانت الإجابة "نعم" أرجو المتابعة:**

4. **ما هو مصادر المعرفة حول وسائل منع الحمل في حالات الطوارئ**

| **المصدر** | **نعم** | **لا** |
| --- | --- | --- |
| ألجريدة |  |  |
| صديق |  |  |
| فرد من العائلة |  |  |
| التليفزيون أوالراديو |  |  |
| طبيب أو أخصائى لتنظيم الأسرة |  |  |

5. **ما هو التوقيت الصحيح لاخذ حبوب منع الحمل في حالات الطوارئ ؟**

□ أقل من 72 ساعة من الجماع □ أكثر من 72 ساعة بعد الجماع

6. **ماذا يمكنك أن تسأل زوجتك لمنع الحمل؟**

□ اطلب من الزوجة أن تأخذ جرعة عالية من حبوب منع الحمل

□ اطلب من الزوجة استخدام وسائل منع الحمل الطارئة

□ اطلب من الزوجة تركيب جهاز داخل الرحم ( لولب )

□ اطلب من الزوجة الإجهاض

□ اطلب من الزوجة استخدام العلاجات العشبية

□ الدعاء

7. **لماذا تستعمل حبوب منع الحمل الطارئة؟**

□ لمنع الإجهاض □ لمنع الحمل الغير مرغوب فيه □ لتنظيم فترات الحمل

8. **متى يمكن للنساء استعمال حبوب منع الحمل الطارئة؟**

□ لو حدث قطع في الواقى الذكرى

□ نسيان اخذ حبوب منع الحمل

□ فشل قذف المنى بالخارج

□ عدم اللجوء الى طرق الموانع

9. **هل حبوب منع الحمل الطارئة يمكن الحصول عليها؟**

□ نعم □ لا

10. **حبوب منع الحمل الطارئة يمكن الحصول عليها من:**

□ مستشفى حكومي □ مستشفى خاص □ الصيدلية الخارجية

11. **هل يجب ان تستشير طبيب قبل استخدام حبوب منع الحمل الطارئة؟**

□ نعم □ لا

12. **هل يجب الحصول على حبوب منع الحمل الطارئة عن طريق:**

□ نصيحة طبيب □ نصيحة صيدلى □ بدون نصيحة

13. **هل يلزم عمل اختبار حمل قبل استخدام حبوب منع الحمل الطارئة؟**

□ نعم □ لا

14. **هل سبق لك استخدام وسائل منع الحمل في حالات الطوارئ لمنع الحمل في الماضي؟**

         □ نعم □ لا

**الاتجاهات**/المواقف:

15. **هل الإعلان عن حبوب منع الحمل الطارئة يجب ان يكون واسع الانتشار؟**

         □ نعم □ لا

16. **هل يجب الحصول على حبوب منع الحمل الطارئة بدون وصفة طبية؟**

         □ نعم □ لا

17. **هل تشعر بالخجل من طلب وسائل منع الحمل في حالات الطوارئ؟**

□ نعم □ لا

18. **قرار استخدام وسائل منع الحمل في حالات الطوارئ هو في النهاية قرار:**

 □ الزوج □ الزوجة □ كلاهما

19. **هل تفضل أن تحصل زوجتك على وسائل منع الحمل في حالات الطوارئ من الصيدلية أو العيادة؟**

□ نعم □ لا

20. **يجب أن يكون الرجال قادرين على شراء وسائل منع الحمل في حالات الطوارئ**

□ نعم □ لا

21. **وسائل منع الحمل في حالات الطوارئ يقلل من فرصة الحمل بنسبة تصل إلى 75 ٪ ، هل تطلب من**

**زوجتك استخدامها لمنع الحمل؟**

□ نعم □ لا 3

22. **أود شراء وسائل منع الحمل في حالات الطوارئ لتكون بالمنزل أو في متناول اليد، فقط في حالات الطوارئ**

□ نعم □ لا

23. **من شأن الرجال الذين يمكنهم شراء وسائل منع الحمل في حالات الطوارئ أن يساعدوا في منع الحمل غير**

**المخطط له.**

□ نعم □ لا

24. **أوصي الرجل بوسائل منع الحمل في حالات الطوارئ لخطر التورط في الحمل غير المخطط له.**

□ نعم □ لا

**المعرفة:**

**ما هي الحواجز الممكنة أمام شراء وسائل منع الحمل في حالات الطوارئ؟**

25. **الإحراج عند شراء وسائل منع الحمل في حالات الطوارئ**

□ نعم □ لا

26. **إذا لم تكن على استعداد لاستخدام وسائل منع الحمل في حالات الطوارئ ، فما السبب؟**

**يمكنك أختيار أكثر من سبب:**

| **السبب** | **نعم** | **لا** |
| --- | --- | --- |
| أسباب دينية |  |  |
| أسباب طبية كالآثار الجانبية مثل الغثيان والصداع. |  |  |
| الحالة المادية. |  |  |
| صعوبة في الوصول لها. |  |  |
| التكلفة |  |  |

4
