# Supplementary material for: Men’s knowledge, attitude, and barriers towards emergency contraception: A facility based cross-sectional study at King Saud University Medical City
Source: PLoS One. 2021 Apr 26;16(4):e0249292. doi: 10.1371/journal.pone.0249292 (PMC8075244; doi:10.1371/journal.pone.0249292)
Supplement: S2 File — (DOC) [file pone.0249292.s004.doc]

**What do men know about Emergency Contraception Pills? A survey on knowledge, attitudes and barriers towards its use**

**Principal Investigator Name :**

Dr Syed Irfan Karim , Assistant Professor.

**Office Address :**

Dept. of Family & Community Medicine

College of Medicine

P.O.Box 2925 ,Riyadh 11461

Tel : 4670836 - 4677953

Mobile :0565046182

**CONSENT FORM**

*Dear Participant*

*,*

*This survey is part of study addressing “What do men know about emergency contraception? A survey on knowledge, attitudes and barriers towards its use”. The questionnaire is of self-report and anonymous.*

*Emergency Contraception is an important option for couples who wish to avoid pregnancy in case of family planning method failure or unprotected sex. However, due to lack of knowledge and information about the usage of emergency contraceptive pills, they are underutilized. It is important to explore the knowledge and attitudes of men regarding EC, as well as to identify barriers to its use. The information will contribute substantially to interventions needed for dissemination of information to combat unintended pregnancies.*

*Sharing medical knowledge refers to the exchange of information, advice, ideas, reports and scientific discoveries with other physicians in the medical community.*

*Your response would be much appreciated in this important subject. Your participation in this research study is voluntary.* *Your responses will be confidential.* *The results of this study will be used for scholarly purposes only.*

*.****Estimated Time to complete Questionnaire : 7 -10 minutes***

**What do men know about Emergency Contraception Pills ? A survey on knowledge, attitudes and barriers towards its use**

**Questionnaire**

**Age** (yrs)

**Marital status:** □ Married □ Unmarried, □ Divorce

**No of children:**

□ None □ One □ Two □ More than 2 □ More than 5

**Current desire for child** □ YES □ NO

**Nationality:** □ Saudi □ Non Saudi

**Level of education**

□Primary school □ Intermediate school □ High school □ University □ Higher education

**Occupational Status:**

□ Government □ Private □ Retired □ Unemployed

**Monthly Income:**

□ less than SR 5000 □ In between SR5000-10.000 □ More than SR10.000.

**Knowledge:**

1. **Have you ever heard of any following methods of contraception?**

|  | Yes | No |
| --- | --- | --- |
| Condom |  |  |
| IUCD |  |  |
| Oral contraceptives |  |  |
| Injections |  |  |
| Withdrawal methods |  |  |
| Emergency Contraception |  |  |

2. **If a man has unprotected sex, is there anything he can ask his wife to do in the first 3 days after intercourse to prevent pregnancy?**

□ Yes □ No □ Don’t know

3. **Ever heard of Emergency Contraception? (asked to those who say yes to question 1)**

□ YES □ NO

**If THE ANSWER IS YES than proceed:**

**4 . What is the source of knowledge about Emergency Contraception?**

| **Source** | **Yes** | **No** |
| --- | --- | --- |
| Magazine |  |  |
| Friend |  |  |
| Family member |  |  |
| Television and or Radio |  |  |
| Doctor or family planning provider |  |  |

**5.** **What is the correct timing of Emergency Contraceptive Pills?**

□ Less than 72 hours □ More than 72 hours

**6. What can you ask your wife to do to prevent pregnancy?**

□ Ask the wife to take extra birth control pill

□ Ask the wife to use emergency contraception

□ Ask the wife to have an Intra Uterine Device (IUCD) inserted

□ Ask the wife to have an abortion

□ Ask the wife to use herbal remedies

□ Pray

**7.Why would you use Emergency Contraceptive Pills?**

□ To prevent Abortion □ To prevent unwanted pregnancy □ For Birth Spacing

**8. When women can use Emergency Contraceptive Pills?**

□ Condom Break.

□ Forget to take Pill.

□ Failed withdrawal ejaculation.

□ Failure to use barrier methods.

**9. Is Emergency Contraceptive Pills available in the Market ?**

□ Yes □ No

**10. The ECP can be obtained from ?**

□ Government Hospital □ Private Hospital □ Outside Pharmacy

**11. Do you need to consult a doctor before using Emergency Contraceptive Pills ?**

□ Yes □ No

**12. Should Emergency Contraceptive Pills be available?**

□With doctor’s advice □With advice of pharmacist □ Without any advice.

13**. Is pregnancy test required before Emergency Contraceptive Pills?**

□ Yes □ No.

**14. Have you ever used Emergency Contraceptive Pills to prevent pregnancy in the past?**

□ Yes □ No

**Attitudes:**

15. **Should Emergency Contraceptive Pills be more widely advertised?** □Yes □No

16. **Should Emergency Contraceptive Pills be available without prescription?** □Yes □No

17. **Would you feel shy to ask for Emergency Contraceptive Pills?** □ Yes □No

18. **The decision to use ECP is ultimately the decision of:**

**□** Male partner □ Female partner □ Both

**19. Would you prefer your partner to get Emergency Contraceptive Pills from the pharmacy or clinic?**

□Yes □No

**20. Men should be able to buy Emergency Contraceptive Pills?** □ Yes □ No

**21. Emergency Contraceptive Pills reduces the chance of pregnancy by up to 75%, would you ask your wife to use it to prevent pregnancy?** □ Yes □ No

**22. I would buy Emergency Contraceptive Pills to have at home or on hand, just in case of emergencies.**

□ Yes □ No

**23. Men being able to buy Emergency Contraceptive Pills would help to prevent unplanned pregnancies.**

□ Yes □ No

**24. I'd recommend Emergency Contraceptive Pills to a man at risk of being involved in an unplanned pregnancy.**

□ Yes □ No

**Cognition:**

**What are the barriers to purchase Emergency Contraceptive Pills?**

**25. Embarrassment to buy** □ Yes □ No

**26. If you are not willing to use Emergency Contraceptive Pills what is be the reason?**

| **Reason** | **Yes** | **No** |
| --- | --- | --- |
| Religion |  |  |
| Culture |  |  |
| Difficulty to access |  |  |
| Drug Side Effects (like Nausea, Vomiting) |  |  |
| Cost |  |  |
